# Can Physical and/or Sexual Abuse Play a Role in the Female Choice of a Partner? A Cross-Sectional, Correlational Pilot Study

**DOI:** 10.3390/ijerph17186902

**Published:** 2020-09-21

**Authors:** Erika Limoncin, Caterina Solano, Giacomo Ciocca, Daniele Mollaioli, Elena Colonnello, Andrea Sansone, Filippo Maria Nimbi, Chiara Simonelli, Renata Tambelli, Emmanuele Angelo Jannini

**Affiliations:** 1 Chair of Endocrinology and Medical Sexology (ENDOSEX), Department of Systems Medicine, University of Rome Tor Vergata, 00133 Rome, Italy; erika.limoncin@gmail.com (E.L.); caterina-solano@virgilio.it (C.S.); giacomociocca@gmail.com (G.C.); daniele.mollaioli@gmail.com (D.M.); elena_colonnello@hotmail.it (E.C.); andreasansone85@gmail.com (A.S.); 2 Department of Dynamic and Clinical Psychology, “Sapienza” University of Rome, 00185 Rome, Italy; filipponimbi@hotmail.it (F.M.N.); chiara.simonelli@uniroma1.it (C.S.); renata.tambelli@uniroma1.it (R.T.)

**Keywords:** physical/sexual abuse, facial preferences, sexual functioning, sexual dimorphism

## Abstract

The present study aims to evaluate the relationship in women between a history of physical/sexual abuse and the preferences regarding the choice of a partner for a short/long-term relationship in terms of male facial dimorphism, and to assess their sexual functioning. We enrolled 48 abused women and 60 non-abused women. Facial preferences were evaluated with the Morphing test. Sexual functioning was measured with the Female Sexual Function Index (FSFI). Regarding the choice for a short-term partner, abused and non-abused women did not show any differences, and both groups chose a less masculine male face. On the other hand, regarding the choice for a long-term partner, abused women showed a preference for an average male face, whilst non-abused women preferred a less masculine face. The sexual functioning of abused women was found significantly dysfunctional in all domains of the FSFI. These data, generated from a small but highly selected cohort, demonstrated that physical/sexual abuse may be associated with a more rational and conscious choice of a male partner for a long-term relationship, but not with an instinctive one, as the choice of an occasional partner. In addition, the sexual functioning of abused women appears to be compromised by the traumatic experience.

## 1. Introduction

It is well known that physical and sexual abuse can profoundly impact women’s psycho-sexual well-being [1] by provoking long-term negative consequences, such as the development of several psychopathologies [2], substance or alcohol abuse [3], overt sexual dysfunctions [4,5] and suicide [6].

Whilst these problematic aspects may be directly associated to the experience of sexual abuse, they also serve to maintain a vicious cycle, in which the abused woman chooses an aggressive and potentially abusive man. In fact, as suggested by previous studies [7], abused women may have the tendency to continue choosing an abusive man. However, this finding is not unanimously supported by all experts in this field [8]. Although it was evidenced that about 49% of women who experienced sexual abuse in childhood also experience sexual abuse in adulthood [9], it was highlighted that this vicious circle is not always directly associated to sexual abuse, but rather to the consequences that some women develop due to sexual abuse, such as the consumption of drugs and the adoption of sexually risky behaviors, which expose the woman to a higher risk of encountering aggressive male partners [10,11,12,13,14,15]. Other hypotheses regarding the tendency of abused women to choose a violent male partner were related to the theory of inter-generational transmission of the abuse [16], or the development of emotional dysregulation and other maladaptive coping strategies [17]. A process like that associated to the development of specific psychopathological behaviors (i.e., addictions) occurs while a healthy environment, characterized by positive family relationships, involvement and a secure attachment style, appear to be protective factors. In case of sexual abuse, instead, the exposure to an environment characterized by sexual abuse may enhance the probability to perpetrate the same abuse, or to be abused [16].

Therefore, during adulthood, the role of past sexual abuse may condition the woman’s strategies for the choice of a partner. Amongst the strategies, facial preferences represent an important and documented parameter which varies significantly for both males and females [17,18] in accordance with sexual orientation and gender identity. In addition, for females, these choices may be related to hormonal fluctuations during specific phases of the menstrual cycle [19,20,21] or to specific life conditions, such as pregnancy [22]. Together with all these variables, sexual abuse, either directly experienced or witnessed during childhood, may potentially influence the preference for a male aggressive partner.

The tendency to choose a male aggressive partner may appear as paradoxical. Literature in the field of evolutionary psychology demonstrates that dimorphic male faces (i.e., those with exaggerated male characteristics, such as, for example, those with a pronounced jawbone) are perceived as more aggressive, but also as attractive [23]. However, they are also regarded as an expression of good gene quality [24], which has been shown to be positively correlated with higher salivary testosterone concentrations [25,26], and negatively correlated with a higher predisposition toward respiratory infections, antibiotic use and weaker general health [27,28]. Some studies have instead demonstrated that females prefer less masculine male faces [29,30], whereas other studies have shown that females prefer average male faces, i.e., faces with average characteristics (neither feminine nor masculine) [31,32].

A meta-analysis tried to explain these controversial findings considering the type of male pictures shown to the women, in some cases individual male faces, and in other cases composed, computer-manipulated faces [17]. According to these authors, women prefer composed male faces with less masculine characteristics, while a more masculine male face is preferred in the case of individual male faces. Another study [33] demonstrated that, differently from the literature evidences, more masculine male faces are judged as more aggressive and less attractive.

In line with Darwin’s theories of sexual selection [34], the exaggerated traits of a species may confer an advantage during the competition for a sexual partner (intersexual competition) or guarantee for a win over a potential competitor in a same-sex context (intrasexual competition) [35]. Hence, the male dimorphic face, which gives to the woman the possibility to transmit to the offspring the best gene quality, is also the face of an aggressive man, who at the same time potentially jeopardizes the possibility of pregnancy and motherhood. We can speculate that the vicious circle, which makes the abused woman more vulnerable to the choice of an aggressive male partner, may be evidenced also with a subtler strategy, that is the choice, among a series of manipulated photos of less or more dimorphic male faces, of a face of a hypothetical partner for a short- and long-term relationship.

The choice of a potential male partner by abused women remains a controversial or poorly studied topic. However, clearer data are available regarding the negative effects of sexual abuse on female sexual function. As suggested by several authors, sexual abuse is often associated with female sexual dysfunction, more specifically, with difficulties in the desire and orgasm phases [4,5]. What remains unclear is the link between sexual abuse, the development of sexual dysfunctions and the choice of a hypothetical partner. The interest in studying the relationship amongst these three factors combined is due to the fact that sexual dysfunction in abused women may potentially have a role in conditioning their preference towards an aggressive male face.

Based on these assumptions, we hypothesize that women with a history of sexual abuse will choose, for a short- and long-term relationship, a more dimorphic male face with respect to non-abused women. This study hypothesis is measured assessing the difference in the score of the Morphing test between abused and non-abused women. Additionally, the prevalence of sexual dysfunctions among our sample of abused women, together with the evaluation of all the phases of female sexual response, was explored in order to evaluate whether sexual dysfunction may be related to the choice of a partner.

## 2. Materials and Methods

We enrolled 48 women from specific voluntary associations dedicated to psychological support for women with a recurrent history of physical/sexual abuse from childhood to adulthood, and/or having been physically and/or sexually abused in the period precedent to the study enrollment (1–3 months). Additionally, 60 female university students were included as a comparison group. The recruitment strategy of abused women comprised the following criteria: the voluntary willingness to participate in the study and the absence of an excessively recent instance of physical/sexual abuse (<1 month). The recruitment strategy of women without a history of physical/sexual abuse consisted of these criteria: the voluntary willingness to participate in the study and the absence of physical/sexual abuse.

For the condition of “sexual abuse”, we considered both child physical/sexual abuse and adult physical/sexual abuse, sexual assault and sexual harassment. For simplicity, all these conditions will be defined as “sexual abuse”.

### 2.1. Demographics and Clinical Characteristics

We assessed demographic and clinical characteristics with a qualitative questionnaire or an interview.

None of the participants showed overt clinical manifestations of endocrinological diseases. The presence of psychopathologies among abused or non-abused women was assessed by an experienced clinical psychologist during the first two encounters in the support centers and at the University. In both groups, the presence of psychopathologies was assessed by using the Diagnostic and Statistical Manual of Mental Disorders Fifth Edition criteria [36].

In the case of abused women, the occurrence of physical and/or sexual abuse was assessed during an interview, while in the case of non-abused women, this was specifically asked with a question inserted in the qualitative questionnaire (“Have you ever been physically and/or sexually abused?”). Physical and/or sexual abuse were defined using international criteria for the definition of emotional, physical and sexual abuse [37,38,39]. In addition, as previously suggested [40], subjective methods of definition of this phenomenon were used. This subjective method, which comprises a specific question investigating the presence of abuse among adult women who have been recently abused and/or have been abused as a child, was matched with additional criteria in order to better understand the phenomenon. The criterion of a minimum age differential between victim and perpetrator was applied, including in this range both the abuse of the adult and the minor towards the child. In addition, the presence of real or perceived coercion, with a negative reaction by the victim regarding physical contact and/or penetration, was also considered as abuse [41].

### 2.2. Sexual Activity

Sexual activity was evaluated with the Female Sexual Function Index (FSFI) [42]. The FSFI is a well validated and standardized test, composed of 19 items gathering in six domains evaluating female sexual functioning.

### 2.3. Facial Preferences

The subjects were made to choose one picture in line with their preference of a specific male face for a hypothetical short- or long-term relationship, which was evaluated with the Morphing test [22,43,44]. The Morphing test, broadly used in the scientific context [22,43,44], is composed of 21 frontal pictures of male and female faces, which are manipulated with a specific computer program. Facial stimuli were averaged and warped by a procedure identical to that described by Perrett et al. [45]. The two sets of 21 stimuli were assembled in two GIF files and were presented as a QuickTime Movie. The frame number 1 corresponds to the face with the reduced dimorphism, the frame 11 corresponds to the average face and the final frame number 21 shows the face with the enhanced dimorphism. For the statistical purpose, the 21 frames were converted in arbitrary units. To the frames from 1 to 10, we attributed the arbitrary negative numbers ranging from −1 to −0.1. To the frame 11, we attributed the arbitrary value of 0. To the frames from 12 to 21, we attributed the arbitrary positive value ranging from +0.1 to +1. The increments were of 0.1 arbitrary unit. These arbitrary units were used for the specific statistical analysis and were then displayed on the axis ordinates (*y*-axis).

The subjects had to choose only one picture, in line with her/his sexual orientation, for a short-term relationship, and another one for a long-term relationship. The two experimental conditions of the Morphing test, the short- and long-term relationship, were explained to the participants as follows: the hypothetical short-term relationship was defined as a purely coincidental relationship of one night, while the long-term relationship was instead defined as a stable relationship with a stable partner.

### 2.4. Sexual Orientation

Sexual orientation was evaluated with the Kinsey scale [46]. This tool is considered a classification system for the definition of different types of sexual orientation. The scale comprises seven levels of sexual orientation. On the extremes, at point 0 and 6, we can find the categories “exclusively heterosexual” and “exclusively homosexual”, respectively. The average point, the point 3, defines individuals with a bisexual orientation. Other categories are: point 1 (predominantly heterosexual, only occasionally homosexual), point 2 (predominantly heterosexual, but more than occasionally homosexual), point 4 (predominantly homosexual, but more than occasionally heterosexual) and point 5 (predominantly homosexual, only occasionally heterosexual). An additional point, the point X, represents individuals which declare to have no socio-sexual contacts or reactions.

### 2.5. Exclusion Criteria and Ethical Approval

Study exclusion criteria were (i) age < 18 years, an ascertained pregnancy, (ii) overt clinical manifestation of endocrine diseases and/or of severe mental disorders such as psychoses, schizophrenia or major depression and (iii) the use/abuse of recreational or prescription drugs.

The participation in the research was on a voluntary basis, with no monetary compensation for participants. All subjects provided a written, informed consent for the scientific use of their scores before filling out the survey. The protocol was approved by the institutional ethics committee.

## 3. Statistical Analysis

In this pilot study, no formal power-based sample size calculation was performed since no previous data on the facial preferences in abused women were published.

Hence, the sample size calculation was based on the general recommendations of current literature for pilot studies [47,48,49,50] which suggests that the use of a study population ranging from 40 to 80 for each study group is enough for a pilot study. According to Whitehead [36], a sample size per single group of at least 25 subjects was adequate for the assessment of small standardized effect size. Hence, in our study, 48 abused women and 60 healthy controls were studied.

In this study, we hypothesized that women with a history of sexual abuse would choose a more dimorphic male face with respect to non-abused women. As an outcome measure, the difference in the score of the Morphing test between the two study groups was measured with the Mann–Whitney test for not-normally distributed continuous variables or by the Student’s T-test for normally distributed continuous variables. The Shapiro–Wilk test was used to test normality. In order to explore the influence of independent variables (presence or absence of sexual abuse) on the dependent variables (scores of the morphing test or total FSFI score and FSFI sub-domains), controlling the effect for the covariates (demographic and clinical variables), an Analysis of Covariance (ANCOVA) was carried out. In the first step, a regression analysis of the covariate variables on the dependent variables was conducted in order to eliminate the influence of the covariates from the analysis. Then, the unexplained variance in the regression model (the residuals) was subjected to an ANOVA in order to test whether the independent variables still influenced the dependent variables after covariates control. The effect size (ES) (Cohen’s d) related to the difference between the two study groups was computed using Psychometrica, an internet open-source tool (https://www.psychometrica.de/effect_size.html). Cohen describes d = 0.2 as small, 0.5 as intermediate and 0.8 as a large effect.

Dichotomous variables were expressed as absolute or relative frequencies. Difference among the dichotomous variables were determined by χ^2^ test with Bonferroni correction for multiple comparisons. All tests were two-sided and were determined by Monte Carlo significance. Monte Carlo calculation was used as it produces a reliable result, regardless of the size, distribution, sparseness or balance of the data. An alpha error of 5% was required to reject the null hypothesis. Data analyses and graphic presentation were performed with the MedCalc software package (V. 12, Ostend, Belgium).

## 4. Results

### 4.1. Female Facial Preference

The two study groups were composed of females who were either physically and/or sexually abused or not. The demographic and clinical characteristics are listed in Table 1. The main differences between the two groups were found in terms of age, presence of psychopathology, being/have been in psychotherapy, recurrent physical/sexual abuse and menstrual cycle phase. In Table 1, the statistical information with the specific tests used was reported. Based on the Kinsey scale [46], all females declared to be heterosexuals. For the study purposes, women were evaluated for the choice of a male face for a hypothetical short- or long-term relationship and for sexual functioning.

**Figure 1:**
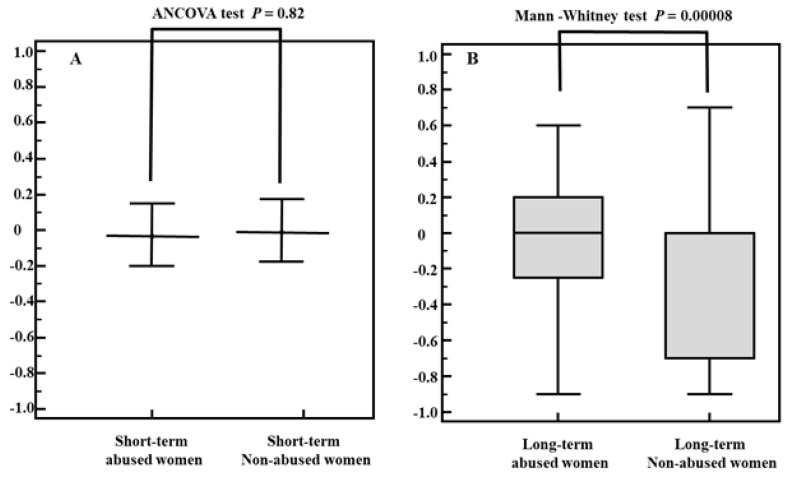
Graphical representation of choices for (**A**) short-term relationship (Mean and CI 95%) and (**B**) long-term relationship (Median and CI 95%) between the two study groups.

In order to evaluate the relationship of the physical and/or sexual abuse with the choice of a possible male partner for a short- or long-term relationship, women were requested to select the most attractive amongst a series of more or less masculine male faces. A regression analysis was performed in order to evaluate which demographic and clinical variables predict the outcome of the dependent variable. In the case of a short-term relationship, the variables age (R_partial_ = −0.27; *p* = 0.0058), FSFI total score (R_partial_ = 0.22; *p* = 0.0243), hormone birth control pill (R_partial_ = −0.38; *p* = 0.0001) and menstrual cycle phase (R_partial_ = −0.23; *p* = 0.0162) significantly influenced the dependent variable. Differently, in the case of a long-term relationship, none of the demographic and clinical variables were retained in the model. Then, the unexplained variance in the regression model (the residuals) was subjected to an ANOVA test for the condition of “short-term relationship”. In Table 2, the adjusted means for covariates, with the F- and the Cohen’s d-values, were reported. The adjusted mean difference between the two study groups in terms of the preference for male face for a short-term relationship did not result statistically significant (F = 0.124; degrees of freedom (df) = 1; *p* = 0.82) with no measurable effect size (ES), (Cohen’s d = 0.069). For what concerns the long-term relationship, the median values were 0 (95% Confidence Interval (CI) −0.19 to 0.03) and −0.38 (95% CI −0.50 to −0.26) for abused and non-abused women, respectively. Using the Mann–Whitney test, a statistically significant difference, with an intermediate ES (Cohen’s d = 0.68), between the two study groups for long-term relationship (Mann–Whitney U value = 901; *p* = 0.0008) was observed. In this case, the choice of abused women for a possible long-term relationship was more oriented for an average, in terms of dimorphism, male face, while the choice of a non-abused women was oriented towards a slightly more feminine face. In Figure 1, the graphical comparison of choices for short- and long-term relationship between the two study groups is shown.

### 4.2. Sexual Activity

A regression analysis was performed in order to evaluate which demographic and clinical variables predicted the outcome of the dependent variable. In the case of total FSFI, the variables recurrent abuse (R_partial_ = −0.22; *p* = 0.026), being/have been in psychotherapy (R_partia l_ = −0.39; *p* < 0.0001) and educational level (R_partial_ = 0.20; *p* = 0.044) influenced dependent variables. The unexplained variance in the regression model (the residuals) was subjected to an ANOVA test for the total FSFI and for its sub-domains. In Table 3, the adjusted means (the residuals), with the F- and the Cohen’s d-values, were reported. The adjusted mean difference between the two study groups in terms of FSFI total score and FSFI sub-domains did result statistically significant. The Cohen’s d values indicated a large ES for both the FSFI total score and FSFI sub-domains, except for the arousal sub-domain (Table 3).

**Figure 2:**
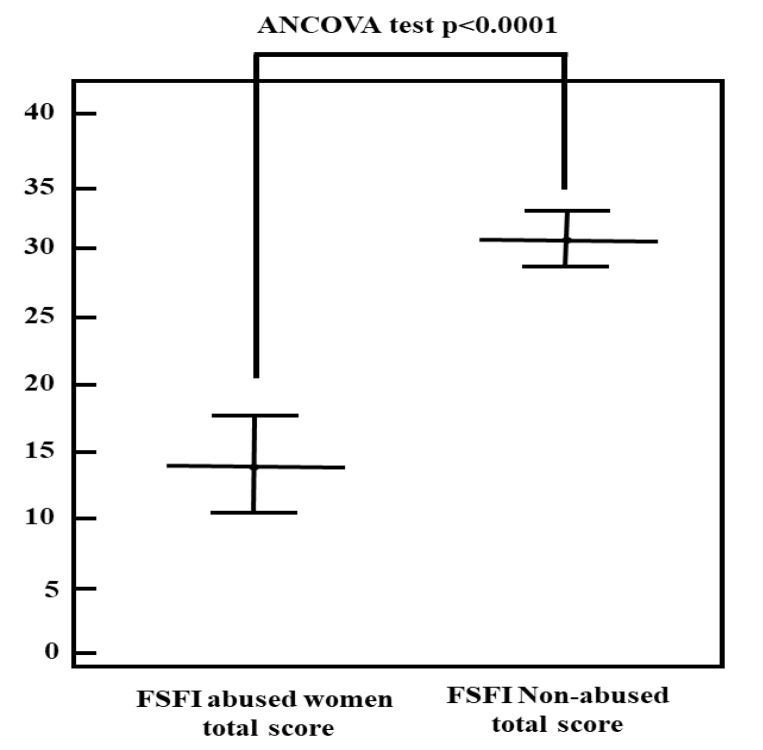
Graphical representation of Female Sexual Function Index (FSFI) total scores between abused and non-abused women (Means and Confidence Interval (CI) 95%).

In Figure 2, the graphical representation of adjusted FSFI total mean scores between abused and non-abused women is shown.

## 5. Discussion

Previous findings have suggested that experiencing violence may have an impact on women’s choices regarding male facial masculinity [51,52,53]. By using a well-validated methodology [22,44], we have explored the association between a history of sexual/physical abuse with the choice of hypothetical male partners for short- and long-term relationships.

Since sexual abuse worsens different aspects of a woman’s life, we hypothesized that being a victim of sexual violence may be significantly related with the choice of a male partner. It has been found that women who feel the fear of being subjected to crime may prefer more masculine traits in the men (in terms of cues of virility, power, strength and ability to succeed in competitions) they choose as partners [54,55]. However, our results depict a more complex scenario. In fact, based on our first analysis, we found a significant difference between the study groups for both short- and long-term relationships. Initially, we found that abused women chose a less dimorphic (more feminized and thus perceived as less aggressive) male face for a short-term relationship compared to non-abused women. This finding led to the hypothesis that abused women might partially change their strategies in facial preferences, showing a less typical strategy compared to that of non-abused women. However, after adjusting for covariates that resulted significant with the multiple regression analysis, this difference was not statistically significant. Hence, although literature suggests that abused women have the tendency to perpetuate the vicious circle of abuse, preferring an aggressive partner [56], in the case of the choice of a male partner for short-term relationship, it seems that sexual abuse could not be directly related with the perpetuation of the risk of abuse. On the contrary, we found a statistically significant difference between the abused and non-abused women regarding the choice of male face for a long-term relationship. In this case, abused women showed a preference for an average (neither too masculine nor too feminine) male face in comparison to non-abused women, who preferred, slightly, but significantly, a more masculine male face. This difference remained significant also after adjusting for covariates, which have been evaluated as relevant with the multiple regression analysis. However, the Analysis of Covariance (ANCOVA) demonstrated that none of the variables inserted in the model could explain this difference. We can hypothesize, in line with previous studies [8], that sexual abuse could be associated with the choice of an average face (neither masculine nor feminine), in terms of masculinity, for a long-term relationship. Hence, in our study sample, it seems that sexual abuse may be associated with the conscious, rational choice of a male partner for a long-term period. Vice versa, we can speculate that sexual abuse does not have the power to weight on the unconscious, emotional and more instinctive choice of a male for a short-term relationship, allowing the woman to follow her innate strategy to choose a less masculine, and less aggressive face, as a guarantee for her own protection and that of her hypothetical offspring. Future research is needed to confirm this interpretation of our data.

It has been demonstrated that mating strategies and facial preferences are influenced by hormonal changes during the menstrual cycle [22,44,57]. During the ovulatory phase, women seem to prefer a more dimorphic face, as a guarantee of good gene quality [21]. Regarding our data, the variable “menstrual cycle phase” was not associated with differences between the two groups regarding the choice of a long-term partner. A theory by Penton-Voak [58] demonstrated that heterosexual female preference for a more dimorphic (i.e., more masculine) male face is determined by the intention to choose better genes (which are thought to be associated with pronounced testosterone-dependent traits of male faces). However, this theory has been partly revised; in fact, some studies show that the tendency for a female to choose a more dimorphic male face can only be confirmed during the ovulatory phase, and only in females who are in a stable relationship [45].

Other discordant findings stated that some females develop a modified version of the above-cited tendency to choose more dimorphic male faces, demonstrating a preference for less dimorphic faces during the ovulatory phase [59]. This shift may be the result of evolved strategies in facial preferences, which may derive from the necessity to provide the offspring with good caregiving, rather than good genes. Other studies have stressed the role of short- or long-term relationships in determining female mating strategies [22,44]. This suggests that, beyond the sexual orientation, gender identity or hormonal status [22,44], women choose, for both short- and long-term relationships, less dimorphic (more feminized) faces, with only small, not statistically significant differences among them.

Whereas female preferences in terms of male facial dimorphism still appear as controversial, the relationship between a history of sexual abuse and female sexual well-being is well established. Our main goal was to verify if all the phases of the female sexual response may be altered by a history of sexual abuse.

The interest in studying the sexual functioning of abused women may shed a light on two possible mechanisms: on the one hand, physical/sexual abuse, as other traumatic events, may profoundly affect sexual functioning [60,61]. On the other hand, the presence of sexual dysfunctions in abused women may be related to the choice of a possible partner. Based on our data, we can affirm that sexual abuse is positively associated with facial preferences of females. Vice versa, we demonstrated that the choice of a possible male partner is not explained by the presence of sexual dysfunctions. Surely, further research is needed in order to fully comprehend this complex relationship.

In accordance with existing literature [62,63,64], we found a statistically significant difference between the two study groups in terms of presence of sexual dysfunctions. In fact, abused women showed a significantly lower, i.e., worse, FSFI score compared to non-abused women. None of the variables inserted in the ANCOVA analysis, which could explain this difference following the multiple regression analysis (the use of hormonal birth control pills, and the “being/have been in psychotherapy”), resulted significant. Hence, we can suppose that in our case, the difference in FSFI scores between the groups may be attributed to the presence of sexual abuse. In addition, differently from literature data, which suggest an impact of sexual abuse prevalently on the sexual desire and sexual arousal FSFI sub-domains, in this study, a statistically significant difference in all the five FSFI sub-domains was found, suggesting that sexual abuse may be associated with all the phases of the female sexual response. This data may be considered during clinical practice, in order to build the best sexological treatment, respectful of the patient/couple and of their needs.

Studying the facial preferences and sexual functioning of women who have experienced sexual abuse is correct and appropriate, but, at the same time, quite difficult, considering the intimate link between sexuality, the choice of a potential partner and the sexual abuse itself. These difficulties have subjected our study protocol to some methodological limitations, which may impact the generalizability of findings. First, our data must be considered cautiously, because this study represents an attempt to assess the relationship between sexual abuse and the female preferences for male face in an indirect manner. Another limit is represented by the testing of the hypothetical partner preference rather than the actual partner preference. Related to this first aspect, there is also the limit represented by the choice of a comparison group, that is a group of female university students. The choice of an appropriate comparison group for an observational study may be in some cases considered a problem. In our case, this group differs in terms of some demographics. However, we tried to mitigate this bias using advanced statistical approaches demonstrating that this limit did not impact on the principal findings. In addition to this, we have planned another study investigating the partner choice strategies of abused women with a larger population sample. Thirdly, the non-generalizability of our data may be partly due to the fact that about half of the sample of abused women has to be considered as “symptomatic”, due to the presence of a recent physical/sexual abuse. Hence, our data must be considered as a snapshot of the sexual and “relational” functioning during a traumatic experience. Finally, the absence of specific follow-ups does not allow us to fully understand how sexual abuse is associated with the female facial preferences and sexuality through time. Future research should also consider longitudinal evaluations specific to this field.

## 6. Conclusions

Our preliminary data suggest that sexual abuse is related to the way females choose their potential partners for a long-term, stable relationship, inducing them to prefer, contrary to the gender-dependent mating strategy, an average male face, which means a face with an average dimorphism. In addition, the choice of a possible male partner seems not to be explained by the presence of female sexual dysfunctions, inducing us to suppose that there is an exclusive link between the choice of a partner for a long-term relationship and the sexual abuse. As evidenced by our findings, the experience of sexual abuse implies a worsening in all the phases of sexual response, and not only on the sexual desire, as previously suggested. These data may contribute to the understanding of this phenomenon and of its relationship with the female relational and psycho-sexological aspects. Specifically, the study of facial preferences and of their potential changes, for example during/after psychotherapy, might aid in monitoring the therapeutic outcome, that is the prevention of future sexual abuses, thus providing a potential useful tool for psychologists and healthcare professionals.

## Figures and Tables

**Table 1:** Demographic and clinical characteristics of the study population.

Variables	Abused Women (N = 48)	Non-Abused Women (N = 60)	Mann–Whitney U and χ^2^ Values	Cohen’s d	*p* *
**Age**					
(median; 95% CI)	34.5; (31.7 to 36)	25; (23 to 27)	697.5 (Mann–Whitney U)	0.985	0.001 *
**Education (%; N)**					
Junior high school	22.9; 11/48)	8.3; (5/60)	3.413 (χ^2^)	0.361	0.65
Senior high school	52; (25/48)	43.3; (26/60)	0.506 (χ^2^)	0.137	0.48
University degree	25; (12/48)	48.3; (29/60)	5.20 (χ^2^)	0.449	0.023 *
**Marital status (%; N)**					
Single	52; (25/48)	35; (21/60)	2.49 (χ^2^)	0.307	0.14
Cohabitant/Married	22.9; (11/48)	56.7; (34/60)	11.18 (χ^2^)	0.7	0.001 *
Divorced	8.3; (4/48)	8.3; (5/60)	0.123 (χ^2^)	0.067	0.72
Widowed	14.6; (7/48)	0; (0/60)	9.96 (χ^2^)	0.637	0.016 *
**Hormonal contraceptive pills**					
(estro-progestinic) (%; N)	18.7; (9/48)	25; (15/60)	0.302 (χ^2^)	0.106	0.58
**Menstrual cycle phase**					
Pre-ovulatory (%; N)	27; (13/48)	48.3; (29/60)	6.59 (χ^2^)	0.509	0.012 *
Post-ovulatory (%; N)	73; (35/48)	51.6; (31/60)	0.891 (χ^2^)	0.182	0.345
**Psychopathology (YES)**					
(%; N)	22.9; (11/48)	0; (0/60)	10.82 (χ^2^)	0.667	0.001 *
**Being/have been in psychotherapy (YES)**					
(%; N)	64.6; (31/48)	5; (3/60)	41.19 (χ^2^)	1.57	<0.01 *
**Recurrent sexual/physical abuse from childhood to adulthood**					
(YES) (%; N)	52.1; (25/48)	0 (0/60)	37.8 (χ^2^)	1.467	<0.01 *

* *p* < 0.05, * *p* < 0.01.

**Table 2:** The Analysis of Covariance (ANCOVA) of short-term relationship with adjusted means weighted for the covariates and Cohen’s d effect size.

Abused Women (n = 48) (Adjusted Means and CI 95%)	Non-Abused Women (n = 60) (Adjusted Means and CI 95%)		
−0.009 (−0.20 to 0.18)	0.024 (−0.14 to 0.19)		
Post-hoc pairwise comparisons: abused vs. non-abused women			
Adjusted mean difference −0.03371	Standard error 0.1550	F value (df) Cohen’s d 0.124 (1) 0.069	*p*-value 0.82

F = F-test; df = degrees of freedom.

**Table 3:** ANCOVA analysis of Female Sexual Function Index (FSFI) sub-domains and total score with adjusted means weighted for the covariates and Cohen’s d effect size.

FSFI Domains	Abused Women (n = 48) (Adjusted Means and CI 95%)		Non-Abused Women (n = 60) (Adjusted Means and CI 95%)	
Desire	2.69 (2.3 to 3.1)		5.53 (5.2 to 5.8)	
Arousal	3.1 (2.5 to 3.7)		4.8 (4.3 to 5.3)	
Lubrication	2.6 (1.9 to 3.2)		4.9 (4.4 to 5.5)	
Orgasm	2.4 (1.8 to 3.1)		4.8 (4.2 to 5.3)	
Satisfaction	2.4 (1.8 to 3.1)		5.4 (4.8 to 5.9)	
Pain	1.6 (1.1 to 2.1)		5.7 (5.2 to 6.1)	
Total score	14.1 (11.2 to 17)		31.2 (28.7 to 33.7)	
	Post-hoc pairwise comparisons: abused vs. non-abused women			
	Adjusted mean difference	F value (df)	Cohen’s d	*p*-value
Desire	2.84	87.278 (1)	1.83	<0.001
Arousal	1.68	12.764 (1)	0.70	<0.001
Lubrication	2.36	21.904 (1)	0.92	<0.001
Orgasm	2.35	21.424 (1)	0.91	<0.001
Satisfaction	2.93	37.819 (1)	1.20	<0.001
Pain	4.10	112.369 (1)	2.07	<0.001
Total score	17.1	57.041 (1)	1.48	<0.001

F = F-test; df = degrees of freedom.
